# Creating space to talk about patients’ personal goals: experiences from primary care stakeholders

**DOI:** 10.1186/s12875-022-01956-9

**Published:** 2023-01-14

**Authors:** Dagje Boeykens, Reini Haverals, Muhammed Mustafa Sirimsi, Lotte Timmermans, Dominique Van de Velde, Patricia De Vriendt, Pauline Boeckxstaens, on behalf of the Primary Care Academy, Roy Remmen, Emily Verté, Peter Van Bogaert, Hans De Loof, Kris Van den Broeck, Sibyl Anthierens, Ine Huybrechts, Peter Raeymaeckers, Veerle Buffel, Dirk Devroey, Bert Aertgeerts, Birgitte Schoenmakers, Lotte Timmermans, Veerle Foulon, Anja Declerq, Nick Verhaeghe, An De Sutter, Lies Lahousse, Peter Pype, Ann Van Hecke, Peter Decat, Rudi Roose, Sandra Martin, Erica Rutten, Sam Pless, Vanessa Gauwe, Didier Reynaert, Leen Van Landschoot, Maja Lopez Hartmann, Tony Claeys, Hilde Vandenhoudt, Kristel De Vliegher

**Affiliations:** 1 Department of Rehabilitation Sciences, Occupational Therapy, Faculty of Medicine and Health Sciences, Ghent University, Ghent, Belgium; 2 Department of Public Health and Primary Care, Faculty of Medicine and Health Sciences, Ghent University, Ghent, Belgium; 3 Centre for Research and Innovation in Care, Faculty of Medicine and Health Sciences, University of Antwerp, Antwerp, Belgium; 4 Department of Primary Care and Interdisciplinary Care, Faculty of Medicine and Health Sciences, University of Antwerp, Antwerp, Belgium; 5 Academic Centre of General Practice, KU Leuven, Louvain, Belgium; 6 Department of Occupational Therapy, Artevelde University of Applied Sciences, Ghent, Belgium; 7 Frailty in Ageing (FRIA) Research Group, Department of Gerontology and Mental Health and Wellbeing (MENT) Research Group, Faculty of Medicine and Pharmacy, Vrije Universiteit, Brussels, Belgium

**Keywords:** Patients’ goals, Patient preferences, Chronic care, Chronic conditions, Multimorbidity, Primary care stakeholders, Primary care, Patients’ goals, Qualitative research

## Abstract

**Background:**

To address the many challenges health systems and communities face, primary care is constantly searching for new strategies to improve quality of care. One of the strategies is to focus on patients’ personal goals to direct the care process. To adopt an explicit focus on patients’ personal goals, actions at different levels are required. As a first step in this process, this study aims to explore the experiences of primary care stakeholders (i.e., scholars, primary care providers, and policy makers) and develop a comprehensive understanding on the idea ‘putting patients’ goals first’. This will help to formulate suggestions about what these actions should include.

**Method:**

In this study, 41 primary care stakeholders participating in six focus groups between January 2020 and September 2020, were recruited via maximal variation purposive sampling. Data collection was done through an open-ended semi-structured interview guide. Focus groups were audio-recorded, transcribed verbatim, and analyzed following a phenomenological-hermeneutical philosophy of Lindseth and Norberg.

**Results:**

All participants expressed a strong fundamental belief for putting patients’ personal goals first. The primary care providers shared that they created space for patients’ personal goals by letting them talk about their values and stories. They reported to integrate their medical expertise with patients’ personal goals in order to develop a balanced relationship. In this context, they also talked about the importance of taking into account the perspectives of patients’ significant others. Primary care providers also talked about how they used patients’ personal goals as a guide in interprofessional collaboration. Scholars denoted that (future) care providers need more training to acquire competencies to discuss patients’ personal goals. The providers and policy makers talked about organizational limitations in terms of time restrictions and the lack of registration systems to support a workflow oriented towards patients’ personal goals.

**Conclusions:**

This study can be used to support the coherence of the development of different actions and strategies to get primary care stakeholders fully on board to support the adoption of patients’ personal goals in care delivery at different levels. However, models of practice and policy plans are needed to work towards a person-centered integrated system.

**Supplementary Information:**

The online version contains supplementary material available at 10.1186/s12875-022-01956-9.

## Introduction

Primary care is defined as “an integrated, accessible system by health and welfare providers who deal with a large majority of personal health care needs, developing sustained partnership with patients, and practicing in the context of family and community” [1]. In the context of the increasing number of people living chronic and complex care needs (i.e., “patients”) the importance of a primary care system cannot be understated [1]. Primary care is the setting where care for these patients mostly takes place, and care plans are developed. These care plans, often including an overload of medication prescriptions, referrals, etc. can make patients feeling overwhelmed and might result in fragmented care [2, 3]. The treatment burden reported by these patients raises the question on how primary care should be best transformed to better meet their needs and improve their quality of care and life [4].

To do so, the World Health Organization (WHO) recommends a shift towards person-centered integrated care (PC-IC) with a focus on patients’ personal goals [5, 6]. A person-centered health system on the one hand builds on empowering people to participate in their care process, tailoring care delivery to their needs, and valuing the input and support of informal caregivers [7]. Integrated health services, on the other hand, “manage and deliver care in a way that ensures people receive a continuum of health promotion, disease prevention, diagnosis, treatment, etc. at the different levels and institutions of care within the health system, and according to their needs throughout their life course" [5].

Following these WHO global recommendations, the Flemish primary care system (Dutch-speaking part of Belgium) is transforming to better support primary care practices, and foster intersectoral and interprofessional collaboration. One of the core strategies in this primary care reform is to explicitly direct care on the patients’ personal goals [8]. To support this transformation, different organizations are established to link policy and practice, to translate governmental objectives into actions, and set up new partnerships among all primary care stakeholders. An example of such governmental organization, is the Flemish Institute of Primary Care (FIPC) that develops policy plans and strategies for local primary care settings [9]. Besides this example, other projects were established through public financial support such as the Primary Care Academy, funded by the King Baudouin Foundation. The Primary Care Academy focusses on primary care research and its implementation in education [10]. More locally, practice-based projects have emerged to strive for better coordination and cooperation between individual primary care stakeholders and organizations and promote a focus on the patients’ personal goals [8]. The common ground of all these initiatives is the aim to develop strategies or interventions to pursue a focus on patients’ personal goals [4].

A focus on patients’ personal goals is hypothesized as a catalyst for developing a person-centered and integrated health system [11]. It might also be worthwhile to direct care on patients’ personal goals as it seemed to improve the social well-being, physical well-being, and satisfaction of people with chronic conditions and from those who deliver care, and thus improve their quality of care [7, 12–14]. Despite the relevance and potential of putting patients’ personal goals first, there is still a knowledge gap about the actions needed to facilitate this transition in primary care [15]. These actions could be situated in the field of research, practice, and policy and should result in well-designed models of practice or interventions and policy plans [4]. As a first step in this process, it is important to incorporate viewpoints of all primary care stakeholders (scholars, primary care providers, and policy makers) into a comprehensive understanding of the idea “putting patients’ personal goals first”. Therefore this study aims to explore the experiences of scholars, primary care providers, and policy makers to formulate suggestions about what these actions should include.

## Method

### Research team

This study was conducted by an interprofessional research team embedded in the Primary Care Academy. This study has been performed by a team of occupational therapists, pharmacists, general practitioner, and gerontologist. This interprofessional approach ensures a diverse and broad perspective when analyzing the data of primary care stakeholders.

### Design

This study used a qualitative, phenomenological-hermeneutical method of Lindseth and Norberg [16, 17]. This approach is suited to enable a greater understanding on the (lived) experiences of primary care stakeholders (scholars, primary care providers, and policy makers) regarding the phenomenon under investigation: experiences of putting patients’ goals first, the strategies they use, and the challenges they encounter [18]. To collect data, a focus group methodology was used. These focus groups were organized in four waves characterizing an iterative process; the new questions that arose from each wave preceding another, guided the interview guide of the consecutive focus groups until data saturation was obtained.

The entire method of this study is checked with the Consolidated criteria for Reporting Qualitative research (COREQ) (see Supplementary File 1) [19].

### Participants and sampling

The participants represented a broad variation of scholars, primary care providers, and policy makers with experience and expertise in primary care; named as the primary care stakeholders. They were contacted via the network of the Primary Care Academy (phone and mail) using a maximum variation sampling strategy following the principles of Patton 2014 [20].

Focus groups were conducted in four waves. The first wave comprised one pilot focus group with scholars and members of the Primary Care Academy consortium. The second wave included three parallel focus groups with primary care providers who, based on their patient contact, could discuss how they put patients’ goals first during their patient encounters. In a third wave, one focus group with a selection of participants of wave two took place to validate or invalidate the results of the previous waves. For this focus group, the selection of participants was based on professions and their contribution and engagement during the focus groups of wave two. In the final and fourth wave, scholars and policy makers were selected when they adopted patients’ personal goals in they research, development of tools, or taking it as a leading principle in their work.

Contributing to the focus groups was completely voluntary and participants did not receive any form of renumeration.

### Data collection

The focus groups (January 2020-September 2020) were coordinated by a moderator (DB, MMS, or LT) trained in qualitative research techniques.

For each wave, a semi-structured interview guide with open-ended questions was tailored based on the results of the previous waves. Topics relating to the phenomenon under investigation were discussed in depth. The first wave aimed to explore the topic broadly allowing adaptations to the interview guide for the following waves. The second wave allowed more in-depth information about their experiences regarding patients’ personal goals, how they enabled patients to manage their conditions, and how they fostered collaboration. Specific topics for this second wave were ‘understanding on putting patients’ personal goals first, ‘adoption of patients’ personal goals’, ‘implementation of patients’ personal goals’ in their practice. For the third wave, the preliminary results were presented to broaden insights on specific topics (e.g., ‘tension that providers might experience when putting patients’ personal goals first’). Based on the first three waves code saturation was reached meaning that no new themes were identified. A fourth wave was organized to deepen the data and get full understanding of the themes which allowed to reach meaning saturation [21]. If necessary, additional questions or probes were used in all focus groups to encourage the participants to clarify and elaborate on their answers. The moderator was supported by an assistant who wrote down the non-verbal observations, kept an eye on the time, passed on additional questions, or opened the floor to someone. Focus groups took place at the university or online due to the Covid19 measures.

### Data analysis

The focus groups were audio-taped and transcribed verbatim. The phenomenological-hermeneutical approach described by Lindseth and Norberg was used [16]. This method consists of three steps: 1) naïve understanding, 2) structural analysis, and 3) comprehensive understanding. The analyzing process from writing down the naïve understanding to the comprehensive understanding is characterized by an iterative process. Throughout this process, specific attention was given to distinct between the participants’ different backgrounds and illuminate their lived experiences. This was done by allocating meaningful units and quotations to the corresponding participant and referring to their specific input while analyzing towards to the different themes.

The naïve understanding is ‘a first conjecture of the analysis and has to be validated or invalidated by the more extensive structural analysis’ [16]. The initial naïve understanding was formulated after intensive reading of the transcripts from the first two waves and was written in such way that the text speaks for itself. This initial naïve understanding was written by the first authors (DB, RH), consistently discussed with the team, and finally rewritten by the research team (DB, RH, DVdV, PDV, PB). In the structural analysis meaning units, condensations, sub themes, and themes were identified and described as condensed descriptions to illuminate lived experiences [16]. In a first phase of this structural analysis, the first two focus groups were analyzed to elicit preliminary themes. These preliminary results were then presented and discussed in the interdisciplinary context of the researchers (occupational therapists, pharmacists, general practitioner, and gerontologist). Additionally they were presented to externals with the aim to validate the results and identify remaining questions. In a second phase of the structural analysis, the four remaining focus groups were analyzed with the preliminary themes kept in mind to generate new themes. Saturation was reached as no new themes arose. In a third phase of the structural analysis, the themes were revisited again until consensus by all co-authors was achieved before they were written down in final themes. Finally, the comprehensive understanding was created with the aim to critically reflect on the relation between the themes, the research question, the study context, and finally formulate the participants’ lived experiences as a whole. For this, the transcripts were read again with the naïve understanding and structural analysis in mind. This stepwise approach allowed to become more familiar with the data and gain in-depth knowledge in the lived experiences of primary care stakeholders regarding the research aim.

### Ethical approval

Ethical approval was obtained from the Ethical Committee of University of Antwerp (B300201942302). The study was in accordance with the principles outlined in the Declaration of Helsinki. The participants received verbal and written information about the purpose and methods of the study. They all gave written informed consent. No incentives were given to the participants.

## Results

A total of 41 primary care stakeholders participated in six focus groups. An overview of the participants is given in Table 1. They presented a broad range of disciplines working in primary care ensuring a multidisciplinary character of the focus groups. The focus groups lasted between 68 and 150 min.

**Table 1:** Overview of the participants

Focus group	Code	Discipline	Job description
Wave 1 (123 min)	P1	Gerontologist & speech therapist	Policy worker (PM)
	P2	Nurse	Professor (S)
	P3	Pharmacist	Professor (S)
	P4	Physiotherapist	Teaching assistant (S)
	P5	Occupational therapist	Professor (S)
Wave 2a (68 min)	P1	Social worker	Care coordinator (PM)
	P2	Psychologist	Lecturer (S)
	P3	Nurse & Diabetes educator	Project worker (PM)
	P4	Nurse	Diabetes nurse (P)
	P5	Pharmacist	Pharmacist (P)
	P6	Social worker	Director of patient organization (PM)
	P7	Pharmacist	Pharmacist (P)
Wave 2b (90 min)	P1	Social worker	Social worker (P)
	P2	Pharmacist	Pharmacist (P)
	P3	General practitioner	General practitioner (P)
	P4	Sociology & Healthcare management	Policy worker patient organization (PM)
	P5	Pharmacist	Pharmacist (P)
	P6	Pharmacist	Pharmacist (P)
	P7	Psychologist	Psychologist (P)
	P8	Pharmacist	Pharmacist (P)
Wave 2c (119 min)	P1	Occupational therapist	Occupational therapist (P)
	P2	Occupational therapist	Expert vulnerable populations (P)
	P3	Social worker	Social worker (P)
	P4	Nurse	Home nurse (P)
	P5	Nurse	Care coordinator (PM)
	P6	General practitioner	General practitioner (P)
	P7	Occupational therapy	Lecturer (S)
	P8	Social worker	Manager non-residential care (PM)
Wave 3 (107 min)	P1	Psychologist	Psychologist (P)
	P2	Nurse & Diabetes educator	Project worker (PM)
	P3	General practitioner	General practitioner (P)
	P4	Sociology & healthcare management	Policy worker patient organization (PM)
	P5	Social worker	Social worker (P)
	P6	Social worker	Manager non-residential care (PM)
Wave 4 (86 min)	P1	Psychologist	Consultant (PM)
	P2	Biomedical sciences	Project leader (PM)
	P3	Social worker	Policy worker (PM)
	P4	Social pedagogy	Director home care organization (PM)
	P5	Nurse	Lecturer (S)
	P6	General practitioner	Professor (S)
	P7	Gerontologist & speech therapist	Policy worker patient organization (PM)

*Abbreviations*: *S* Scholars, *P* Primary care providers, *PM* Policy makers

Given that the sample represent a diversity of primary care stakeholders, distinctions between their different professional backgrounds have been described in the results to illuminate their different lived experiences.

### Naïve understanding

The initial reading of the transcripts revealed that all participants have a strong belief that it is worthwhile to focus on patients’ personal goals. The primary care providers put patients’ personal goals first by talking about things that matters most to them and addressing their personal goals and values. They experienced a better understanding of the patients’ situation during their encounters by doing so. If they were aware of the patients’ personal goals, they felt they were better placed to inform the patients about treatment options allowing them to make well-considered decisions regarding their goals. In this context, all participants talked about the importance of taking into account the goals of patients’ significant others All participants also talked about how they used patients’ personal goals as a guide in interprofessional collaboration. They reflected that this supported the alignment of different care plans of care providers into a shared vision. Despite their overall support towards putting patients’ goals first, participants reported about challenges. The scholars stressed the importance of acquiring competencies by training and education to discuss patients’ personal goals and step aside from thinking in terms of solutions to patients’ problems. The providers and policy makers also talked about organizational limitations in terms of time restrictions and the lack of a registration system to support a workflow oriented towards patients’ personal goals.

### Structural analysis

After selecting and condensing the meaning units, and further analysis, six themes could be elicited from the transcripts (Table 2). Table 3 provides an excerpt from the structural analysis (more information about analysis in Supplementary File 2).

**Table 2:** Overview of the themes

Awareness of the necessity to create time and space to elicit patients’ personal values and stories
Experiencing a balanced relationship through combining own expertise and patients’ personal goals
Taking into account the perspectives of significant others
Feeling connected with other team members through the patients’ personal goals
The challenge to become competent in discussing patients’ personal goals
Experienced organizational limitations

**Table 3:** Example of structural analysis of a meaning unit

Meaning unit	Condensation	Themes
“I think if patients can make an informed choice and say ‘I don’t want to do this’, we as a provider, have to respect that. We have goals we set and patients have their goals. If they don’t agree after they are well-informed about their options, then we have to agree with that (P3 -wave 3)	You have to respect the informed choices the patients make. We can set goals, but if patients do not agree with them, it’s ok	Experiencing a balanced relationship through combining own expertise and patients’ personal goals

#### Theme 1: awareness of the necessity to create time and space to elicit patients’ personal values and stories

All participants expressed a strong fundamental belief that putting patients’ personal goals first was a promising strategy for doing good for patients. According to the primary care providers (e.g., wave 2 and 3), putting patients’ goals first felt as their “natural” attitude. They said that they tried to “create space” to let patients articulate their personal values and stories. During conversations with their patients, participants experienced that they were able to identify things that mattered most to the patients, could gain insight into their needs, and into that what is valuable for experiencing a good life. The scholars and policy makers (e.g., wave 1 and 4) also reflected that it is the intrinsic nature of care providers to put patients’ personal goals first. They acknowledged that it was important to have openness to talk about what matters most to patients.

"When you let people talk about what matters to them, what's important to them, that creates an open door to what matters to them… to values and emotions for what those people find relevant to live a good life, what matters to them, then I feel healthy." (P3, wave 1).

The primary care providers reported that these values gave rise to formulating patients’ personal goals. Besides eliciting goals throughout patients’ stories, they also shared that they explicitly asked questions as “What do you want to achieve?” (P5—wave 3) to elucidate goals. By focusing on patients’ personal goals, participants experienced that they gained more insight into the patients’ lives aside their disease. It gave them the feeling of contributing to the patients’ quality of life.

#### Theme 2: experiencing a balanced relationship through combining own expertise and patients’ personal goals

The primary care providers, reported that they informed patients about different treatment options. They mentioned that it was important for them that their patients were well informed about how procedures could contribute to their quality of life. This was also pinpointed by the policy makers who reported that treatments should be regarded in the light of pursuing quality of life as defined by the patients. By informing patients, they hoped that patients were given the opportunity to make well-considered decisions regarding the goals they wanted to strive for.

"The goals can help to review and look together at the pros and cons of each treatment and see how that matches up with the life and quality of life that the patient sees for himself." (P1, wave 1).

Primary care providers experienced that, by informing patients, they could bring in their own medical expertise into the conversations with their patients, which seemed important to them. They shared that they tried to combine their medical goals with patients’ personal goals into one shared care plan while still respecting patients’ personal goals throughout this process. By equally integrating each perspective, these participants got the feeling of contributing to a balanced relationship.

#### Theme 3: taking into account the perspectives of significant others

The primary care providers felt the need to involve patients’ significant others (e.g., informal caregivers, family, and friends) during consultations as it allowed them to gain more insight into the patients’ living situation. The policy makers (e.g., director, care coordinator) denoted that it was important to acknowledge each individual expertise (e.g., care provider, patient, significant other) as equal because they all had a contributing and supporting role in the care process.

"For me, the core is the equality of everyone involved in the process and from there, going on a journey, each from their own expertise. The physician has his own expertise, the patient has his own expertise, the informal caregivers as well. To identify that equality and bring it together.” (P4 – wave 4).

By exploring the goals of patients’ significant others, all participants experienced that they could unravel their unexpressed frustrations or concerns (“My father wants to drive his car despite his Parkinson’s disease” (P4, wave 1)). Especially in the event that patients set goals that were conflicting with the goals of their significant others, participants expressed that it was important to generate discussion about everyone’s goals. This resulted, based on the participants’ experiences, in less misunderstanding between patients and their significant others. If succeeding in aligning conflicting goals, the primary care providers felt more connected with their patients and their significant others.

"If you then also start to determine the goals of the people surrounding the patient, then suddenly a new world opens up because then you start to see why the daughter is so panicked or asking to have her mother fixated, why her mother becomes so aggressive… And if you then have identified both goals, then you can start to connect and make a compromise of which in the best case they say 'Ah yes, that's why', that’s what brings people closer." (P7, wave 2c).

#### Theme 4: feeling connected with other team members through the patients’ personal goals

All participants shared that patients’ personal goals could be used as potential guidance to organize interprofessional collaboration. The primary care providers shared that they used patients’ personal goals as a way to find connection and to guide team collaboration. The policy makers, reported that they proposed a focus on patients’ personal goals to their teams as a strategy to better collaborate and allocate team members who could best support the patients to work towards those goals.

"…Maybe it can start from the patient, the patient's perspective and then it gives an opportunity for care providers to align with that and then look at it like ‘Okay, what expertise can I add to this?'." (P1-wave 1).

The primary care providers experienced that this focus helped them to get everyone on the same page. The participants shared that they used different ways to integrate patients’ personal goals during interprofessional collaboration. In some cases, goals were discussed in a one-on-one conversation between one provider and the patient prior to interprofessional meetings and were then represented by that provider. In other cases, depending on the organizational structure, patients attended interprofessional meetings and could share their goals in person.

#### Theme 5: the challenge to become competent in discussing patients’ personal goals

All participants experienced that for discussing patients’ personal goals specific competencies needed to be acquired. Participants working in education, observed that most (future) care providers still have the desire to provide strategies and solutions for the patients’ problems instead of focusing on their goals. These participants recognized that competencies need to be acquired during education to be able to shift from a coaching role to a focus on patients’ personal goals.

“Last year on the exam, I acted as a patient with cancer and tiredness. I strongly felt and observed that students adopted a coaching role and relied on their theoretical knowledge instead of integrating all what they have learned to approach me as a person. You have to know when to apply what and to which patient. That require competencies.” (P5 – wave 4).

In addition, the primary care providers experienced that it is hard to always focus on patients’ personal goals. Particularly in cases where the patient did no longer had any goals it felt counterintuitive for them to focus on their goals. In these cases, they shared that they tried to put themselves in place of their patients and wonder if they would be happy with the way how care was delivered.

"I think it's a mind shift for everyone. We as care providers have been way too much of rescuers. All disciplines, always solving things for people, going to rescue them, figuring things out for them, and that's an attitude where that we, we need to step away from that." (P2 -Wave 2c).

#### Theme 6: experienced organizational limitations

The participants were positive towards putting patients’ personal goals first but all shared they encountered organizational limitations for doing so. The main concern primary care providers and policy makers shared was the lack of time to fit discussions on patients’ personal goals into their daily practice. They denoted that, however their organization acknowledged the benefits of a focus on patients’ personal goals, the current structure did not allow them to adopt this focus. However, participants experienced that a focus on patients’ personal goals could save time on the long term as care was, in their opinion, better aligned among the team members and patients were feeling more satisfied.

"…that health care providers don't have the time to fill that out with the patient… Because actually all that time that is put into life goals is seen as kind of lost time. Actually that's free, working for free during that moment. But we do agree that when we look at the greater goal and when we constantly look at the same goal from that focus from different care providers, we're going to obtain a better outcome." (P4-wave 2b).

The primary care providers and policy makers talked about the need for a workflow and registration system that supports providers to start the care process from the patients’ perspectives. One of the suggestions made by, was to acknowledge a goal-setting consultation as an intervention. They stated that also the financing system is still too much focused on pay for performance and number of interventions instead of patients’ personal goals. Changes to this system could support them to reorient their focus. For them, a first step in pursuing a focus on patients’ personal goals is to train and educate management and make them familiar with the potential of this care approach.

### Comprehensive understanding

**Fig. 1:**
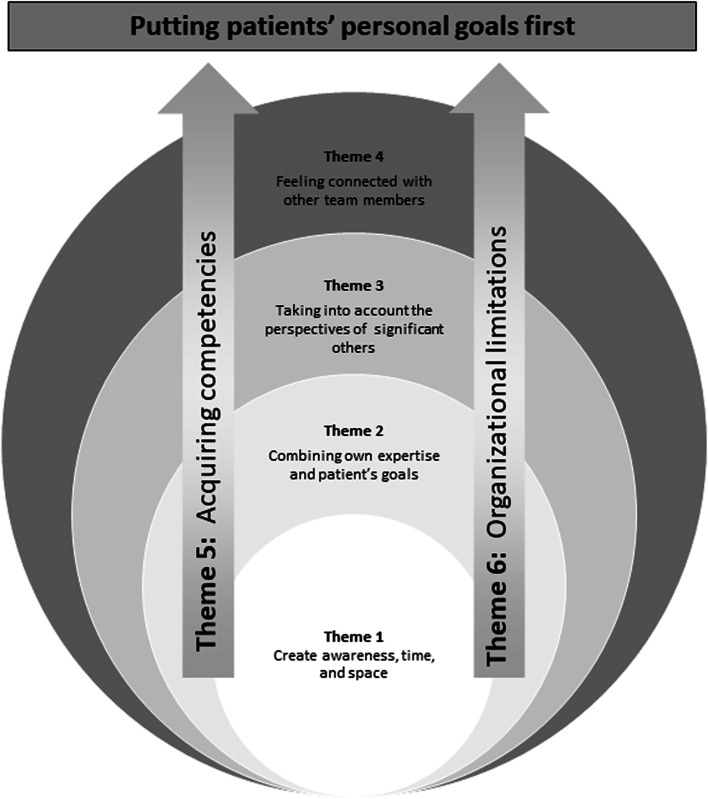
Illustration of the comprehensive understanding

To formulate the comprehensive understanding, the naïve understanding and the themes were re-read, critically reflected on, linked to each other, and considered as an overarching whole to make the patterns between these parts visible. Figure 1 illustrates this comprehensive understanding.

It seemed to be an intrinsic attitude to leave room to explore the patients’ values and stories (theme 1) and at the same time to integrate the medical perspective by sharing treatment options (theme 2). In essence it is important to bridge between the lived experiences of patients and the expertise of providers and translate the patients’ goals into a feasible care plan. The gap that therefore has to be tackled is nourished by the participants’ needs for more competencies to discuss patients’ personal goals first (theme 5) and the organizational challenges they are experiencing in doing so (theme 6). The need for balance between what is important for the patient and for the provider can be further extended to the level of involving the patients’ informal and formal network to generate a care plan tailored to the patients’ personal goals (theme 3). In general and at all these levels, patients’ personal goals were used as a guidance to create a common understanding on how care delivery should be organized (theme 4).

## Discussion

This study examined the experiences of primary care stakeholders towards putting patients’ personal goals first, the strategies they use, and the challenges they encounter when doing so. The structural analysis resulted in six themes of which four of them described main strategies. First, the participants reported that they intrinsically created space towards their patients to explore what is meaningful for them. Second, they informed patients about the treatment options to combine their own medical expertise to patients’ personal goals and strive for the best quality of life as defined by the patient. Third, they listened to the patients’ significant others to elicit their goals and value everyone’s role during the care process. Fourth, they integrated patients’ personal goals during interprofessional collaboration. However, to engage patients in sharing their goals and being able to put the patients’ goals first, the participants encountered two main challenges. In the field of education, focus should be put on equipping (future) providers with the needed competencies to discuss patients’ personal goals. In the field of policy, organizational transformations have to occur prior to sustainable putting patients’ goals first.

Our interview data showed that primary care providers had an intrinsic attitude to put patients’ personal goals first by exploring their values and that what is meaningful for them. However it is not straightforward to inherently align care plans with patients’ personal goals [22, 23]. Weir et al. for instance interviewed general practitioners (GPs) about their perspectives on discussing patients’ personal goals and preferences in regard to medicine management [22]. They found different patterns in GPs practices ranging from ‘considering goals as a low priority’ to ‘goals are central’, indicating that not every GP puts this high on his agenda [22]. Looking further into the literature, Ospina et al. observed how patients’ agendas were elicited during clinical encounters [23]. They reported, contrasting with our findings, that only in half of the primary care encounters the patients’ agenda was elicited and in the other half the providers interrupted their patients too early in their story [23]. As the providers’ experiences contrast with observable results, it can be discussed if providers indeed intrinsically focus on patients’ personal goals or just have the feeling they do.

Reflecting further on the finding that the participants implicitly focused on the patients’ personal goals, it can be questioned if there is indeed a focus on the patients’ personal goals (e.g., ‘I want to visit my overseas friends’) instead of a direct focus on the treatment decision, named in the literature as health-related goals (e.g., ‘I don’t want to take my medication because they make me tired’). The literature bears this doubt and describes that providers tend to go more along with the patient’s health-related goals instead of their personal goals [24–26]. Whether there is a focus on personal or health-related goals, our findings and the literature describe that goals should be placed in the context of the patients’ values and primarily focus on improving the patients’ quality of life [27, 28]. Therefore, patient-provider dialogue is important to integrate those both perspectives and formulate questions as ‘How will these test results help achieve the patients’ goals of care?’ [27, 28]. Raising awareness of primary care stakeholders about these differences is important as a focus on personal goals illustrates the shift away from the traditional, disease-oriented paradigm [29, 30].

Another aspect that was mentioned by the primary care stakeholders, was the importance of involving the patients’ significant others in developing the care plan. Based on the literature, this social environment could play a crucial role in supporting patients to better deal with their complex situation resulting in better prognoses, stress relieve, etc. [31]. We would have expected that by discussing everyone’s goals and roles conflicts could arise, but neither our results or the literature points to that. So described Kaldjian that a shared understanding could be formulated when goals are explained or negotiated [28]. Despite the benefits of involving significant others such as the informal caregiver, it is unclear how that should be organized [32]. This unclarity was also shown in our results as there seemed to be no uniform way to do so. However, when the roles of patients and informal caregivers are recognized within a team setting, it helps the providers to better understand the perspectives of patients and informal caregivers [32]. It might also make the patients feel more supported and satisfied [33, 34].

Despite the eagerness and willingness of the primary care stakeholders to put patients’ personal goals first, they reported the need to develop competencies to do so. A recent scoping review explored the needed knowledge, skills, and attitudes primary care providers must have to deliver person-centered and integrated care. They concluded that the current literature is lacking information about what competencies are needed and how they should be acquired [35]. In any case, primary care providers should be better equipped to make the shift from medical oriented goals towards the patients’ personal goals [36]. To succeed, other concepts in which patients’ personal goals are one of their central pillars might be inspiring. A concept wherein patients’ personal goals get a prominent position is goal-oriented care. In goal-oriented care, care is organized around the patients’ personal goals [30]. It is a promising concept to explore further in regard to the role of patients’ personal goals in care delivery [30]. Also narrative-based medicine might be inspiring as providers try to bridge the gap between the patients’ story and their own story by exploring fears, feelings, and emotions to develop a deeper understanding on their patients [37]. But again, it is not clear which competencies should be developed to deliver goal-oriented care or narrative-based medicine [38]. All findings considered, there can be concluded that more attention has to be given to identifying and developing the needed competencies to integrate patients’ personal goals into care delivery. This should already be addressed in higher education but, once more, the knowledge of how to integrate this into existing programs is lacking [35]. Fortunately, the future looks bright as several initiatives are being taken to strengthen primary care such as the capacity building of primary care research (e.g., Primary Care Academy).

These initiatives – in practice and in education – are important as one of the major barriers is situated at the policy level. Therefore, the policy makers are important stakeholders to get on board to support the adoption of putting patients’ personal goals first [36]. One of the recommendations the participants made, is to acknowledge goal-setting as a full-scale intervention so primary care providers will be rewarded for their time investment. Only in this way, the needed models of practice and policy plans could be developed to work towards a person-centered integrated care system [4].

### Strengths and limitations

Some strengths and limitations go along with the choice for a focus group methodology. First, we have chosen for a phenomenological-hermeneutical design which could seem odd for analyzing focus groups as it aims to deepen lived experiences of individuals which is more related to in-depth interviews. Though, Bradbury et al. described that it is a well-suited design for focus groups as individual lived experiences could also be captured within a group context and it is even beneficial to stimulate discussion and open new perspectives [18]. Nevertheless, it was quiet a challenge to capture the lived experiences of the different participants which could have led to a more abstract description of the themes rather than a description of their lived experiences. However, we have tried to nuance between the different participants’ professional backgrounds and their lived experiences in the description. Second, the participants in our study represented a broad range of primary care stakeholders going from primary care providers to scholars. These different views were an added value to enrich the discussions and gain a representative sample of all people involved in primary care. Moreover, this broad range helped to generate a common understanding on the idea of ‘putting patients’ personal goals first [39]. Third, the different waves not only allowed to deepen the results, but also increased credibility among them and assured data saturation which was achieved in the fourth wave as no new information emerged. In this way the results of any previous wave were also validated. Fourth, this study was conducted by a team of researchers with different backgrounds (occupational therapists, general practitioner, pharmacists, and gerontologist) which reduced the risk of interpretation bias as personal opinions and previous knowledge could not prevail. A reflective attitude was the pillar of the analysis.

### Suggestions for future research

This study adds knowledge on how patients’ personal goals are put first in primary care in Flanders and provides important information to take into account when outlining future research. Future research should focus on identifying the needed competencies to put patients’ personal goals first and translate these competencies into guidance or training packages. The premise of this guidance should be that primary care stakeholders learn how to make the shift from the disease-oriented paradigm to a focus on patients’ personal goals. Besides this education -oriented research, research should also focus on integrating the patients’ perspectives into an understanding on how their goals are addressed during consultations. Therefore, it would be interesting to bring all stakeholders including patients, primary care providers, policy makers, and scholars together to generate a shared understanding on putting patients’ personal goals first by means of a participatory action research.

## Conclusion

The six focus groups with primary care stakeholders provided an answer on what their experiences are regarding putting patients’ personal goals first, the strategies they therefore use, and the challenges they encounter. The results showed that they all expressed a strong belief for putting patients’ personal goals first. They experienced that it is not only a valuable approach during one-on-one consultations, but that it might also be beneficial to reduce misunderstanding among patients and their significant others. Patients’ personal goals seemed also to be used as a guide in interprofessional collaboration as it might have the potential to integrate different care plans with each other. However, the participants mentioned the need to acquire competencies to discuss patients’ personal goals by for example training, and argue for changes at the policy level. Those latter points of attention could nourish future research.

## The Primary Care Academy

Roy Remmen^4^, Emily Verté^4,8^, Muhammed Mustafa Sirimsi^3,4^, Peter Van Bogaert^9^, Hans De Loof^10^, Kris Van den Broeck^4^, Sibyl Anthierens^4^, Ine Huybrechts^4^, Peter Raeymaeckers^11^, Veerle Buffel^12^, Dirk Devroey^8^, Bert Aertgeerts^5^, Birgitte Schoenmakers^5,13^, Lotte Timmermans^5^, Veerle Foulon^14^, Anja Declerq^15^, Nick Verhaeghe^16^, Dominique Van de Velde^1,6^, Pauline Boeckxstaens^2^, An De Sutter^2^, Patricia De Vriendt^1,6,7^, Lies Lahousse^17^, Peter Pype^2,18^, Dagje Boeykens^1,2^, Ann Van Hecke^2,19^, Peter Decat^2^, Rudi Roose^20^, Sandra Martin^21^, Erica Rutten^21^, Sam Pless^21^, Vanessa Gauwe^6^, Didier Reynaert^22^, Leen Van Landschoot^23^, Maja Lopez Hartmann^24^, Tony Claeys^25^, Hilde Vandenhoudt^26^, Kristel De Vliegher^27^.

^8^Department of Family Medicine and Chronic Care, Faculty of Medicine and Pharmacy. Vrije Universiteit Brussel, Brussel, Belgium.

^9^Workforce Management and Outcomes Research in Care, Faculty of Medicine and Health Sciences, University of Antwerp, Belgium.

^10^Laboratory of Physiopharmacology, Faculty of Pharmaceutical Biomedical and Veterinary Sciences, University of Antwerp, Belgium.

^11^Department of Sociology, Faculty of Social Sciences, Faculty of Social Sciences, University of Antwerp, Belgium.

^12^Department of Sociology; centre for population, family and health, Faculty of Social Sciences, University of Antwerp, Belgium.

^13^Department of Public Health and Primary Care, Faculty of Medicine, KU Leuven, Leuven, Belgium.

^14^Department of Pharmaceutical and Pharmacological Sciences, Faculty Pharmaceutical Sciences, KU Leuven, Leuven, Belgium.

^15^LUCAS-Centre for Care Research and Consultancy, Faculty of Social Sciences, KU Leuven, Leuven, Belgium.

^16^Research Group Social and Economic Policy and Social Inclusion, Research Institute for Work and Society, KU Leuven, Belgium.

^17^Department of Bioanalysis, Faculty of Pharmaceutical Sciences, Ghent University, Ghent, Belgium.

^18^End-of-Life Care Research Group, Faculty of Medicine and Health Sciences, Vrije Universiteit Brussel and Ghent University, Ghent, Belgium.

^19^University Centre of Nursing and Midwifery, Faculty of Medicine and Health Sciences, University of Ghent, Belgium.

^20^Department of Social Work and Social Pedagogy, Faculty of Psychology and Educational Sciences, University Ghent, Belgium.

^21^Expertise Centre Health Innovation. University College Leuven-Limburg, Leuven, Belgium.

^22^E-QUAL, University College of Applied Sciences Ghent, Ghent, Belgium.

^23^Department of Nursing, University of Applied Sciences Ghent, Ghent, Belgium.

Maja Lopez Hartmann.

^24^Department of Welfare and Health, Karel de Grote University of Applied Sciences and Arts, Antwerp, Belgium.

Tony Claeys.

^25^LiveLab, VIVES University of Applied Sciences, Kortrijk, Belgium.

Hilde Vandenhoudt.

^26^LiCalab, Thomas University of Applied Sciences, Turnhout, Belgium.

Kristel De Vliegher.

^27^Department of Nursing – homecare, White-Yellow Cross, Brussels, Belgium.

## Supplementary Information

**Additional file 1.**

**Additional file 2.**

## Data Availability

The datasets used and/or analysed during the current study are available from the corresponding author on reasonable request.
