# Supplementary material for: Creating space to talk about patients’ personal goals: experiences from primary care stakeholders
Source: BMC Prim Care. 2023 Jan 14;24:11. doi: 10.1186/s12875-022-01956-9 (PMC9840292; doi:10.1186/s12875-022-01956-9)
Supplement: Supplementary file 2 — Additional file 2. [file 12875_2022_1956_MOESM2_ESM.docx]

**Supplementary File 2: Overview analysis**

|  | **Preliminary themes*** | **Final themes**** |
| --- | --- | --- |
| - Doing good for patients - Natural attitude - Openness to talk - What matters most to patients | Creating space for personal stories | Awareness of the necessity to create time and space to elicit patients’ personal values and stories |
| - Informed patient - Focus on quality of life - Matching goals - Change in perspective | Combining provider and personal goals | Experiencing a balanced relationship through combining own expertise and patients’ personal goals |
| - Attention for context - Acknowledge individual expertise - Bridging between patient and context - Common goals - Connecting through communication | Valuing the role of significant others | Taking into account the perspectives of significant others |
| - Goals as guidance - Collaboration - Identification team members - Team meetings | Integrating patients’ goals in collaboration | Feeling connected with other team members through the patients’ personal goals |
| - Redefined provider role - Stepping aside from problems - Training and education | Acquiring competencies | The challenge to become competent in discussing patients’ personal goals |
| - Time investment - Acknowledgment intervention - Registration method - Financing method | Dealing with organizational challenges | Experienced organizational limitations |

Table 4 * Preliminary themes defined after first three waves after code saturation ** Final themes defined after meaning saturation expressing the participants’ lived experiences [21] The data analysis was conducted on Dutch data, and as such a Dutch code tree was developed. The original analysis is available upon request in Dutch.
